# Elevated prevalence of age-related macular degeneration in a low-income urban primary care setting

**DOI:** 10.1186/s12982-026-01672-0

**Published:** 2026-03-14

**Authors:** Adriana Kaganovski, Alauddin Bhuiyan, Anisha Kasi, Daniel Kamyab, Aliya Grinberg, Sophie Chen, Robert Thomson, Tasin Bhuiyan, Arun Govindaiah, Avnish Deobhakta, Gareth Lema, Katy Tai, Matthew A. Weissman, Leonard Amoruso, R Theodore Smith

**Affiliations:** 1 Department of Ophthalmology, New York Eye and Ear Infirmary of Mount Sinai, 310 E 14th St, NY New York, 10003-4201 USA; 2 SUNY Downstate Health Sciences University, New York, NY USA; 3 iHealthScreen Inc, New York, NY USA; 4 Icahn School of Medicine at Mount Sinai, New York, NY USA; 5 School of Science, The College of New Jersey, Ewing, NJ USA; 6 McGovern Medical School at The University of Texas Health Science Center, Houston, TX USA; 7 Vitreoretinal Surgery, New York Eye and Ear Infirmary of Mount Sinai, New York, NY USA; 8 Maimonides Medical Center, Brooklyn, NY USA

## Abstract

**Background:**

Age-related macular degeneration (AMD) is the leading cause of vision loss among adults over 50. Early detection enables interventions such as Age-Related Eye Disease Study (AREDS) supplementation to slow disease progression, yet AMD remains underdiagnosed in primary care, particularly in underserved populations. This study assessed AMD sample prevalence and its association with social determinants of health in a diverse, urban primary care cohort.

**Methods:**

We performed a post hoc analysis of a cross-sectional study conducted from June 2020 to May 2023 at a hospital-based primary care clinic in New York City (NYC). Patients aged 50–89 underwent nonmydriatic color fundus photography graded independently by two physicians using Beckman Initiative criteria. Median household income by ZIP code served as a proxy for socioeconomic position. Multivariable logistic regression examined associations between AMD and demographic or social determinants of health variables.

**Results:**

Of 393 patients enrolled, 312 were included in the final analysis. Referable AMD was present in 48.4% of participants based on the Beckman classification, representing the sample prevalence within this clinic cohort and exceeding national population-based estimates. Only 4 of 151 AMD cases had been previously diagnosed. Referable AMD prevalence was higher in males than females (56.2% vs. 44.4%) and increased with age. Median household income was significantly lower among patients with referable AMD compared with those without ($72,000 vs. $151,000; *p* < 0.0001). In adjusted models, male sex (OR = 2.00, 95% CI 1.02–4.00) and lower income were strongly associated with referable AMD prevalence; race and age were not.

**Conclusion:**

Undiagnosed referable AMD was highly prevalent within this primary care clinic sample of low-income, urban patients. Integrating affordable retinal screening, potentially aided by artificial intelligence, into primary care may help address vision health disparities driven by social determinants of health.

**Supplementary Information:**

The online version contains supplementary material available at 10.1186/s12982-026-01672-0.

## Introduction

Age-related macular degeneration (AMD) is the most common cause of vision loss in populations of individuals over the age of 50 [1]. Although new treatments are available for exudative AMD (eAMD), they can only stop the exudation, not reverse the existing ocular damage [2]. Early diagnosis identifies patients with intermediate AMD or with late AMD in the fellow eye who may benefit from AREDS-based supplementation; benefit has not been shown for early AMD [3]. Therefore, regular screening and diagnosis of early AMD in primary care settings can help reduce progression to late AMD, either exudative or atrophic.

However, few studies have explored the prevalence of AMD in primary care medical practices. As one point of reference, however, a cross-sectional study conducted in Birmingham, Alabama found that out of 1288 eyes with no AMD diagnosed by their primary eye care physician, 320 eyes or 24.8% were found to have AMD characteristics upon fundus photograph evaluation by the study investigators [4]. The US Centers for Disease Control and Prevention (CDC) norms, for comparison, were 12.6% for individuals aged 40 and over, suggesting a greater need for high quality screening programs for AMD in general [5].

Socioeconomic status (SES) represents an important but underexplored determinant of AMD detection and management. Lower-income individuals are less likely to receive regular eye examinations or access ophthalmologic care, particularly within urban settings where social and structural barriers to healthcare are pronounced. Primary care providers (PCPs) play a critical role in this context, serving as the first point of contact for patients with visual complaints or systemic risk factors associated with AMD. Integrating AMD screening into primary care encounters may therefore improve early detection and mitigate disparities in diagnosis associated with socioeconomic and racial differences.

The purpose of this study was to determine the sample prevalence of referable AMD among a diverse cohort of urban primary care patients, as well as explore the association between AMD and social determinants of health.

## Methods

This study is a post hoc analysis of a cross-sectional study sponsored by the National Eye Institute (NEI) and conducted by iHealthScreen at Mount Sinai Medical Center, New York City. This study involves human participants and was approved by the institutional review board (IRB) of Mount Sinai Medical Center (IRB approval no 18–00787). The study was performed in accordance with the Health Insurance Portability and Accountability Act (HIPAA) and adhered to the tenets of the Declaration of Helsinki. Informed consent was obtained from all participants prior to enrollment.

Between June 2020 and May 2023, patients aged 50 to 89 years presenting for routine care at a hospital-based primary care clinic in lower Manhattan were recruited consecutively. The clinic serves a diverse urban population representative of the local community.

Inclusion criteria included age between 50 and 89 years, ability to provide informed consent, and capacity to undergo non-mydriatic color fundus photography. Exclusion criteria included refusal to consent, inability to cooperate with imaging, poor-quality fundus photographs precluding reliable assessment of AMD, incomplete demographic or ZIP code data, or ocular conditions preventing safe or adequate fundus photography.

Of the approximately 1,150 unique patients aged 50–89 who visited the clinic during the study period, a total of 393 patients consented to participate, of whom 81 were excluded due to incomplete imaging or missing demographic data. Ultimately, 312 patients were included in the final analysis, reflecting a participation rate of approximately 27.1%. Nonparticipation was primarily due to lack of available time or competing appointments. Given the clinic’s high-volume, safety-net setting, socioeconomic and logistical barriers may also have limited participation. To assess representativeness, demographic characteristics (age, sex, and race/ethnicity) were compared between included participants (*n* = 312) and eligible but non-participating patients (*n* = 838) using independent t-tests for continuous variables and chi-square tests for categorical variables. This was a convenience sample drawn from a single hospital-based primary care clinic, therefore, the results represent sample prevalence within this clinic cohort rather than population-based prevalence estimates.

Color fundus photographs were acquired using a nonmydriatic DRSPlus CFP system (DRSPlus iCare, Centervue, Inc, Fremont, CA). Images were anonymized and independently graded by two experienced physicians, with discrepancies resolved by a third senior retinal specialist. Inter-grader reliability was assessed using Cohen’s kappa (κ) statistic to evaluate consistency of AMD classification overall and by category (no AMD, intermediate AMD, late AMD). The third adjudicator’s assessment determined the final classification for any discrepant cases. Patients were classified as having referable AMD, based on the Beckman classification, if fundus images revealed intermediate AMD (large drusen > 125 microns, or pigmentary abnormalities and medium drusen ≥ 63-<125 microns) or late AMD (lesions associated with neovascular AMD or geographic atrophy) [6]. Cases were classified as previously diagnosed if a diagnosis of AMD was documented in the patient’s electronic health record (EHR) problem list or encounter diagnoses prior to participation in this study. Median household income was estimated by ZIP code based on U.S. Census data and utilized as a surrogate measure of SES. Prevalence of referable AMD was analyzed across 5-year age strata (50–54 through 85–89 years). Median household income by ZIP code was categorized into four groups (<$30,000, $30,000–$75,000, $75,000–$120,000, and > $120,000) to reflect meaningful distinctions in SES relative to the NYC cost of living. These brackets were chosen with reference to Area Median Income (AMI) tiers defined by the NYC Department of Housing Preservation and Development (HPD) and the U.S. Department of Housing and Urban Development (HUD). In 2025, HUD designates 50%, 80%, and 100% of AMI for a three-person household in NYC as approximately $72,900, $116,600, and $145,800, respectively. Thus, the $30,000–$75,000 and $75,000–$120,000 ranges approximate low to middle and upper middle income levels, while > $120,000 reflects households earning above the 80% AMI threshold, indicative of higher-income status in the NYC metropolitan context [7].

Univariate logistic regressions were performed for AMD as the dependent variable with each predictor (age, sex, race, and household income) entered separately. Pairwise correlations between age and income were evaluated using Spearman’s ρ. Partial R² values were calculated for each predictor to quantify its independent contribution to model variance.

To assess potential confounding between age, sex, race, and household income, a multivariable logistic regression analysis was performed to estimate the adjusted odds of AMD by demographic and socioeconomic variables. Social determinants of health, including race, sex, income, and age were incorporated into the analysis. Statistical analyses were conducted using R within the Jupyter integrated development environment and employed the “dplyr”, “tidyverse”, “stats”, “broom”, “performance,” and “tableone” packages. Multiple logistic regression was used to evaluate associations between prevalence of referable AMD and social determinants. Lack of collinearity across all covariates was confirmed by VIF scores below 5. Because this was an exploratory, cross-sectional analysis of all available eligible participants, no formal a priori power calculation was performed. The final sample size (*n* = 312; 151 AMD cases) provided sufficient precision for multivariable logistic regression including age, sex, race/ethnicity, and income, meeting the conventional rule of at least 10 outcome events per predictor variable.

## Results

Among 1,150 eligible patients, 312 (27.1%) participated in the study. Comparison between included and non-participating patients demonstrated no statistically significant differences in age (65.17 ± 8.9 vs. 64.8 ± 9.1 years, *p* = 0.45), sex (female: 66.35% vs. 62.93%, *p* = 0.63), or race/ethnicity (White 32.05% vs. 29.67%, *p* = 0.74; Black 23.39% vs. 19.97%, *p* = 0.86; Hispanic/Latino 39.74% vs. 43.82%, *p* = 0.78; Asian 5.45% vs. 6.54%, *p* = 0.84). These findings indicate that the study cohort was broadly representative of the eligible clinic population.

Demographic data regarding study participants is tabulated in Table 1. The study sample population totaled 312 subjects, consisting of 207 females and 105 males. The average age of female participants at the time of imaging was 65 years of age, while for male was 65.5 years of age. Age-specific referable AMD prevalence in our cohort ranged from 39.47% in participants aged 50–54 years to 75.00% in those aged 85–89 years, with an overall prevalence of 48.40% (Table 2). Across most age groups, referable AMD prevalence was higher among males compared to females, with total male prevalence of 56.19% versus 44.44% in females. The highest prevalence was observed among males aged 70–74 years (81.82%) and females aged 85–89 years (100%), although the latter reflects a small sample size of *n* = 2. Prevalence increased modestly with age, with a notable rise after age 70 in both sexes.

**Table 1:** Demographics of sample at Mount Sinai Union Square

Sample characteristic	Number of individuals in sample population (*n* = 312)	95% CI
Male	105 (33.65%)	–
Female	207 (66.35%)	–
Age 50–54 (years)	38 (12.18%)	–
Age 55–59 (years)	65 (20.83%)	–
Age 60–64 (years)	53 (16.99%)	–
Age 65–69 (years)	58 (18.59%)	–
Age 70–74 (years)	42 (13.46%)	–
Age 75–79 (years)	30 (9.62%)	–
Age 80–84 (years)	22 (7.05%)	–
Age 85–89 (years)	4 (1.28%)	–
Asian	17 (5.45%)	2.93–7.97%
Black or African American (Hispanic or Latino)	8 (2.56%)	0.81–4.32%
Black or African American (Not Hispanic or Latino)	65 (20.83%)	16.33–25.34%
Other Race (Hispanic or Latino)	100 (32.05%)	26.87–37.23%
Other Race (Not Hispanic or Latino)	22 (7.05%)	4.21–9.89%
White (Hispanic or Latino)	16 (5.13%)	2.68–7.58%
White (Not Hispanic or Latino)	84 (26.92%)	22.00–31.85%

**Table 2:** AMD prevalence (%) by age and sex at Mount Sinai-Union Square

Age group	Female AMD prevalence (%)	Male AMD prevalence (%)	Total AMD prevalence (%)
50–54	10/28 (35.71%)	5/10 (50%)	15/38 (39.47%)
55–59	19/44 (43.18%)	10/21 (47.62%)	29/65 (44.62%)
60–64	14/32 (43.75%)	12/21 (57.14%)	26/53 (49.06%)
65–69	17/36 (47.22%)	11/22 (50%)	28/58 (48.28%)
70–74	14/31 (45.16%)	9/11 (81.82%)	23/42 (54.76%)
75–79	9/19 (47.37%)	6/11 (54.55%)	15/30 (50%)
80–84	7/15 (46.67%)	5/7 (71.43%)	12/22 (54.55%)
85–89	2/2 (100%)	1/2 (50%)	3/4 (75%)
Total	92/207 (44.44%)	59/105 (56.19%)	151/312 (48.40%)

Inter-grader agreement for AMD classification was substantial, with an overall κ of 0.81 (95% CI 0.75–0.87). Agreement by category was κ = 0.86 for no AMD, κ = 0.77 for intermediate AMD, and κ = 0.79 for late AMD. Discrepant cases (*n* = 28, 9.0%) were adjudicated by a third senior grader before inclusion in the final dataset.

**Fig. 1:**
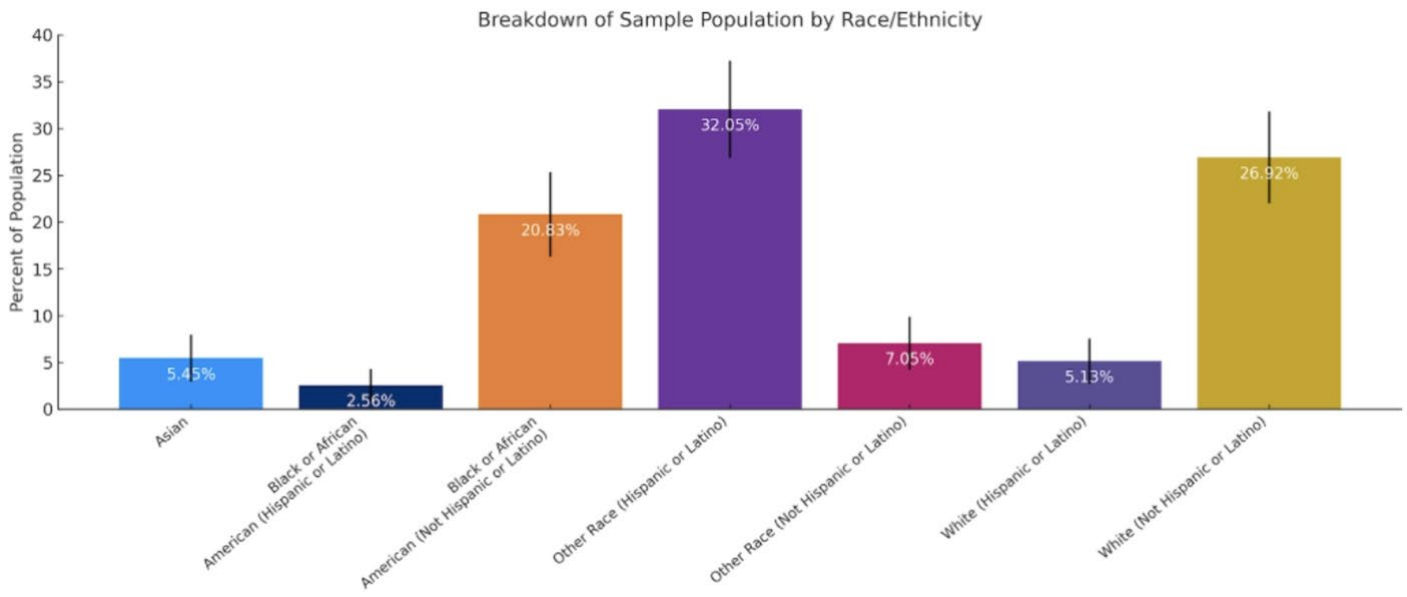
Racial/ethnic breakdown of the study sample population

**Fig. 2:**
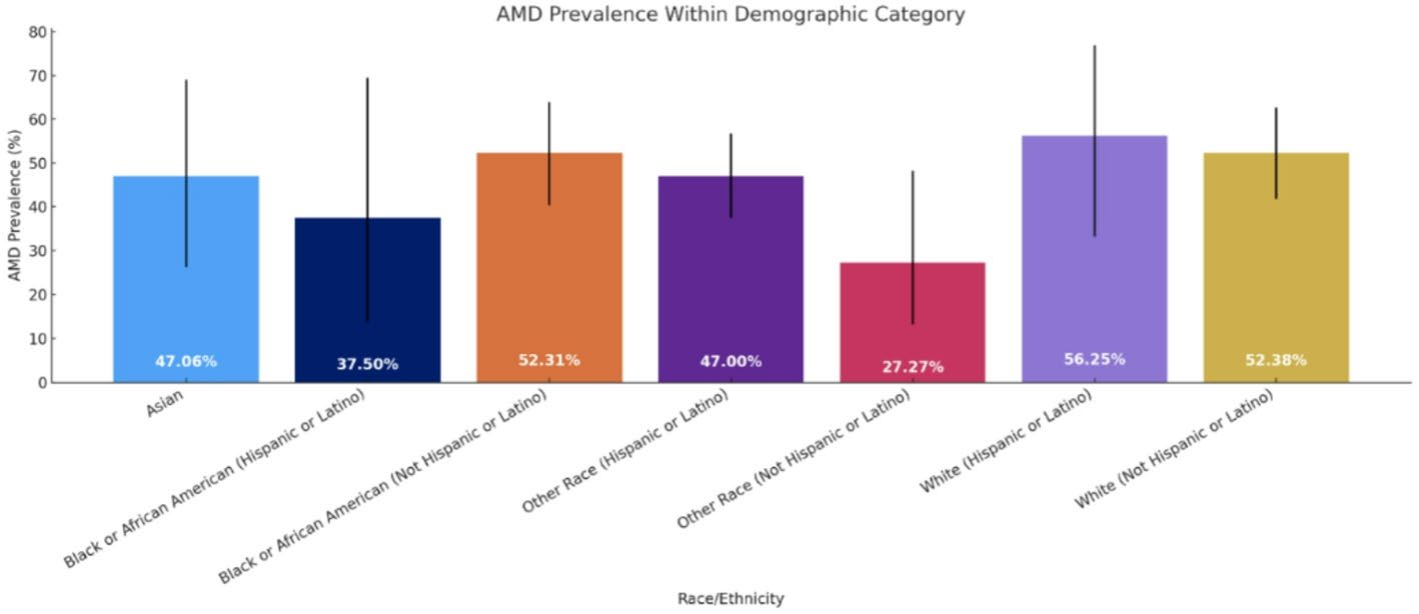
AMD disease burden within each racial/ethnic group

Figure 1 illustrates the ethnic/racial breakdown of participants which consisted of 23.39% identifying as Black or African-American, 5.45% identifying as Asian, 32.05% identifying as White, and 39.1% identifying as “other”. 39.74% of our study participants identified as Hispanic or Latino across all racial categories. No significant differences in referable AMD prevalence were identified in these groups. Table 3; Fig. 2 illustrate the disease burden of AMD within each racial category. The highest prevalences of referable AMD were among Hispanic or Latino Whites (56.25%), non-Hispanic or Latino Whites (52.38%), and non-Hispanic or Latino African-Americans (52.31%). A total of 151 participants were found to have referable AMD. Out of these 151 referable cases, 4 had a prior diagnosis recorded in their EHR. Table 4 summarizes the distribution of disease severity according to the Beckman classification. Overall, 114 participants (36.54%) had no AMD, 47 (15.06%) had early AMD, 134 (42.95%) had intermediate AMD, and 17 (5.45%) had late AMD. Thus, the majority of affected patients had intermediate AMD, with late-stage disease representing a smaller but clinically meaningful subset.

**Table 3:** Demographics of study population and AMD prevalence stratified by racial/ethnic category

Race/ethnicity	AMD prevalence within demographic category	95% CI (Wilson)
Asian	8/17 (47.06%)	26.2–69.0%
Black or African American (Hispanic or Latino)	3/8 (37.50%)	13.7–69.4%
Black or African American (Not Hispanic or Latino)	34/65 (52.31%)	40.4–63.9%
Other Race (Hispanic or Latino)	47/100 (47%)	37.5–56.7%
Other Race (Not Hispanic or Latino)	6/22 (27.27%)	13.2–48.2%
White (Hispanic or Latino)	9/16 (56.25%)	33.2–76.9%
White (Not Hispanic or Latino)	44/84 (52.38%)	41.8–62.7%

**Table 4:** Breakdown of AMD Severity With Proportion of Previously Diagnosed and Undiagnosed Cases

AMD severity	Total cases	Previously diagnosed	Previously undiagnosed
No AMD	114 (36.54%)	–	–
Early AMD	47 (15.06%)	0 (0%)	47 (100%)
Intermediate AMD	134 (42.95%)	3 (2.24%)	131 (97.76%)
Late AMD	17 (5.45%)	1 (5.88%)	16 (94.12%)

**Table 5:**
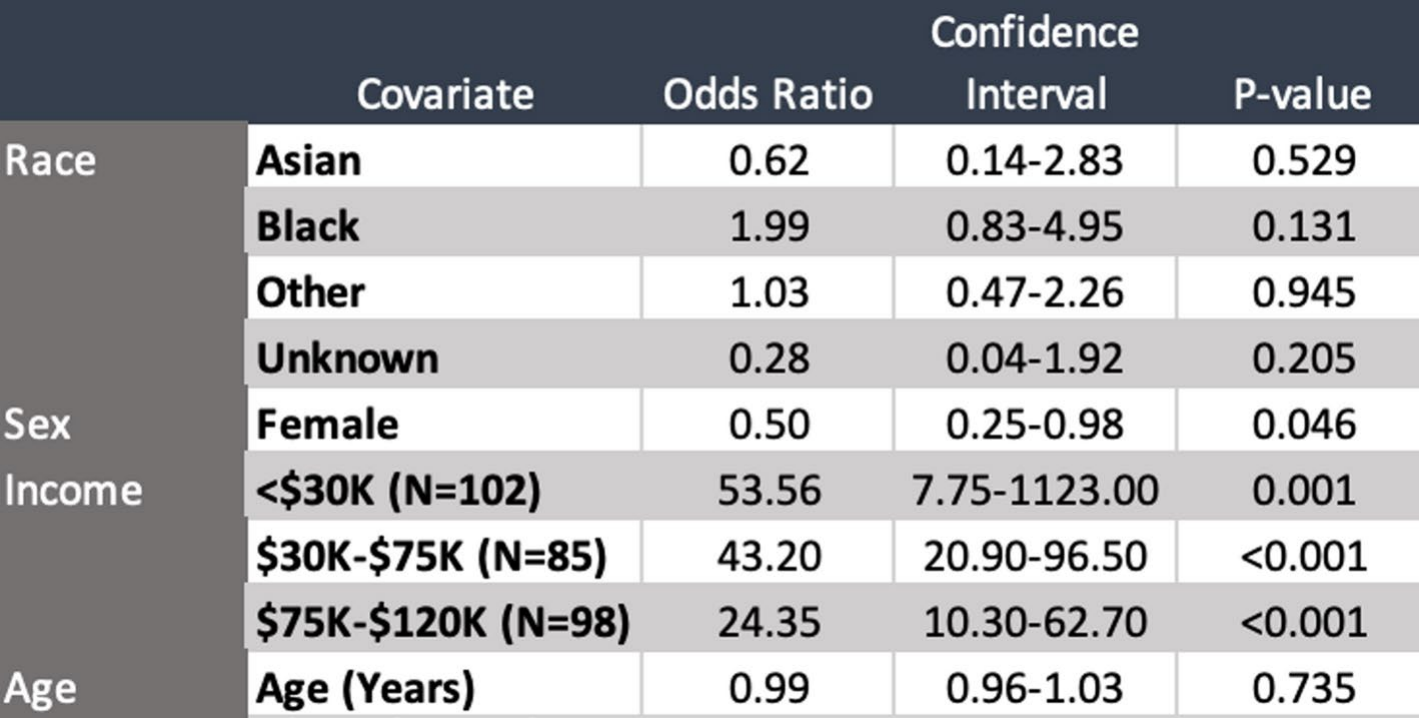
Breakdown of AMD Severity With Proportion of Previously Diagnosed and Undiagnosed Cases

Notably, previously undiagnosed disease accounted for nearly all cases of intermediate and late AMD. Of the 134 patients with intermediate AMD, only 3 (2.24%) had a prior diagnosis, while 131 (97.76%) were newly identified through study imaging. Similarly, 16 of 17 patients with late AMD (94.12%) had no prior diagnosis documented in the electronic health record. All cases of early AMD were newly detected. Undiagnosed AMD was defined as AMD detected on study imaging with no prior AMD diagnosis in the medical record. These findings indicate a high prevalence of clinically significant, previously unrecognized AMD within this primary care population.

**Fig. 3:**
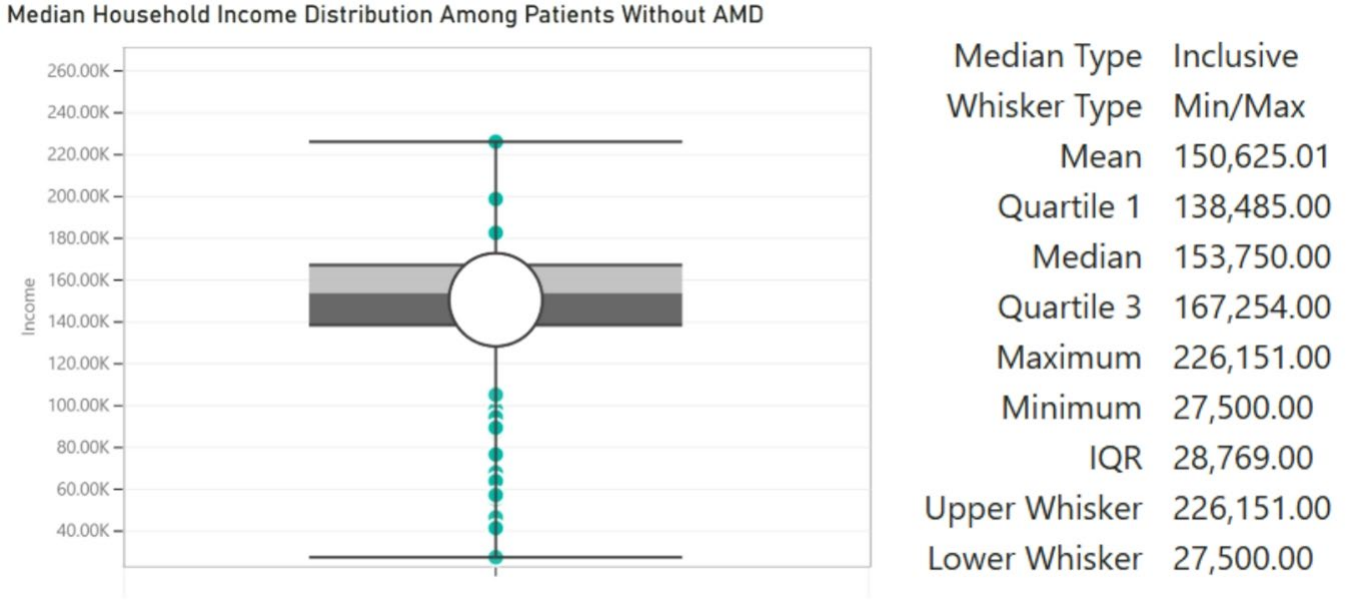
Distribution of household income of patients without AMD

**Fig. 4:**
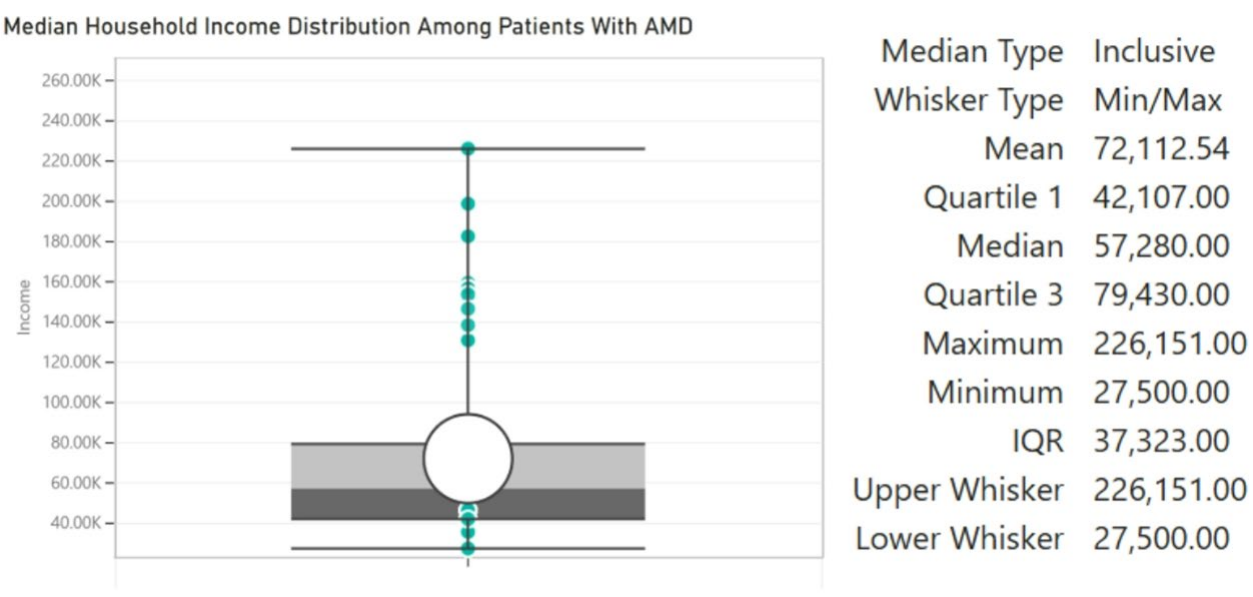
Distribution of household income of patients with AMD

Figure 3 illustrates the average median household income distribution of patients without referable AMD, while Fig. 4 illustrates the average median household income distribution of patients with referable AMD. The prevalence of referable AMD within this cohort was significantly greater amongst patients with lower SES than amongst those with a higher SES. Notably, the median household income of patients with AMD was $72,000 while that of patients without AMD was $151,000 (*p* < 0.0001, two-proportion-z-test). Table 5 presents adjusted odds ratios for referable AMD by race, sex, and household income, using White males with income > $120,000 as the reference group. In multivariable logistic regression analysis, sex and household income were significantly associated with referable AMD prevalence, while race and age were not. Female participants had 50% lower odds of referable AMD compared to males (OR = 0.50, 95% CI 0.25–0.98, *p* = 0.046). A clear dose-response gradient was observed, with progressively higher odds of referable AMD as income decreased relative to the ≥$120,000 reference group. Participants earning <$30,000 had the highest odds (OR = 53.56, 95% CI 7.75–1123.00, *p* = 0.001), followed by those earning $30,000–$75,000 (OR = 43.20, 95% CI 20.90–96.50, *p* < 0.001) and $75,000–$120,000 (OR = 24.35, 95% CI 10.30–62.70, *p* < 0.001). The wide confidence intervals likely reflect the relatively small number of high-income participants, consistent with the clinic’s primarily lower-income population. No statistically significant associations were observed for race or age.

In univariate analyses (Table S1), lower income remained strongly associated with AMD, while female sex was protective. Age and race were not significantly associated. Collinearity diagnostics tabulated in Table S2 indicated no concerning overlap among predictors (VIF 1.03–1.67; Spearman correlation between age and income ρ = − 0.14, *p* = 0.29). Partial R² values were low for most predictors but highest for income (age 0.02, sex 0.05, race 0.04, income 0.21), supporting that income independently contributes most strongly to AMD risk.

## Discussion

Referable AMD was present in 48.4% of participants, representing the sample prevalence within this clinic cohort, which was higher than population-based estimates reported nationally. Strikingly, only four had been previously diagnosed with AMD of any severity, underscoring a substantial burden of undiagnosed disease and critical gaps in access to ophthalmologic screening and preventive eye care within underserved populations.

Previous epidemiologic studies such as the CDC’s Vision and Eye Health Surveillance System and the Beaver Dam Eye Study have reported AMD prevalence rates well below those found in our cohort, with the CDC reporting 12.6% prevalence for individuals aged 40 and over, and the Beaver Dam Eye Study reporting a 17.2% prevalence of early and late AMD across all age groups examined [5,8]. However, those estimates are derived from general population studies, often with less racial/ethnic diversity and fewer participants drawn from clinical care environments. By contrast, our study population reflects a racially and socioeconomically diverse group actively engaged with a safety-net healthcare system, highlighting the real-world disease burden among vulnerable urban populations. It is important to note that different studies may have applied differing AMD grading criteria. Our study followed the standardized AMD classification system proposed by the Beckman Initiative for Macular Research, which aligns with the criteria used in the Alabama and Beaver Dam Eye Study analyses, but may differ from those used by the CDC [6,8]. Additionally, age ranges used for prevalence comparisons differed: our cohort included adults aged 50–89, whereas the CDC reports include individuals aged 40 and older, and the Beaver Dam Eye Study cohort was aged 43–86 [5, 8]. These differences may partially account for the higher prevalence observed in our cohort, as younger individuals are less likely to exhibit AMD.

An additional factor that may contribute to these findings is that our participants were sourced from a primary care setting, whereas both the Beaver Dam Eye Study and the CDC study were general population studies. Primary care patients often come to their PCP to manage chronic conditions, and are therefore likely to suffer from various comorbidities and various degrees of accompanying systemic inflammation. Both chronic systemic disease and systemic inflammation are well described in the literature as risks for AMD [9]. These risk factors generally and our corroborative findings specifically suggest a benefit for more eye screenings in primary care settings.

No statistically significant differences in referable AMD prevalence were observed by race or ethnicity, despite substantial cohort diversity. In adjusted analyses, female sex and socioeconomic status were the primary predictors, with females having 50% lower odds of referable AMD than males. Lower income was strongly associated with higher AMD prevalence in a dose-dependent manner, with the greatest risk among participants earning less than $30,000 and progressively lower odds in higher income brackets.

Although many population-based studies report either small sex differences or higher crude AMD prevalence among women, often attributed to their greater longevity, the elevated risk in men seen in some clinical cohorts, including ours, may have plausible biological and healthcare system explanations [10]. First, sex-specific immune responses and inflammatory regulation differ. Males often exhibit lower immune surveillance and slower resolution of inflammation compared with females [11]. Inflammation is a central driver of AMD pathogenesis; activation of the complement cascade, recruitment of macrophages and microglia, and chronic para-inflammation at the retinal pigment epithelium–choroid interface contribute to drusen formation, photoreceptor injury, and progression to advanced AMD [12,13].

In addition, hormonal and genetic factors shape immune and complement activity, key to AMD biology. Testosterone has been shown to enhance inflammatory signaling pathways, including inflammasome activation, while X-linked immune genes and escape from X-inactivation may afford women immune advantages [14]. Finally, clinical sampling biases, such as men presenting later in disease or with more comorbidities, may amplify observed sex differences in clinic-based studies versus population cohorts. Taken together, these immunologic and healthcare-access factors provide a plausible framework for the elevated AMD odds seen in men in our sample. Future work incorporating AMD subtyping and direct measurement of sex-specific biomarkers would help clarify causality.

The association we found between lower SES and higher referable AMD prevalence reinforces existing literature linking health disparities with poorer outcomes. Patients with referable AMD had median household incomes less than half those of patients without referable AMD, emphasizing how structural inequities, such as barriers to eye care, lack of routine screening, and health literacy challenges, contribute to delayed diagnosis and preventable vision loss [15]. These disparities are particularly concerning given that early-stage AMD is amenable to interventions that can slow disease progression, such as AREDS-based vitamin supplementation or timely referral for monitoring [3]. Yet without targeted screening, many individuals will only be diagnosed at advanced stages when vision loss is irreversible.

Our findings suggest that primary care clinics, especially those serving medically underserved populations, may be pivotal venues for integrating low-cost, non-invasive screening technologies. Prior studies have demonstrated the feasibility and acceptability of using nonmydriatic fundus photography in similar settings [4]. These results suggest a need for large-scale, high-quality screening programs for AMD that could perhaps be facilitated with artificial intelligence (AI). AI-based models for AMD screening with color fundus photos, clinical and socio-demographic data can detect AMD and predict the 2-year risk of developing advanced AMD with 99.2% accuracy [16]. Development and implementation of clinical AI technologies for reliable, timely, and noninvasive screenings of primary care populations would be indicated. Ultimately, this study adds to the growing body of evidence that urban populations with lower SES are disproportionately affected by preventable eye disease. Incorporating vision screening into primary care workflows, and aligning these efforts with broader health equity initiatives, has the potential to reduce disparities in blindness and visual impairment. Further research is warranted to assess implementation models, cost-effectiveness, and patient-centered outcomes of such interventions.

The higher prevalence of referable AMD observed in this study may also be influenced by selection bias, as individuals presenting to urban safety-net primary care clinics may differ systematically from the general population in health status and healthcare-seeking behavior. Limited access to primary and specialty care, particularly ophthalmology, in lower-income areas may contribute to both underdiagnosis and delayed detection of AMD. These disparities in healthcare access and screening awareness could lead to an apparent concentration of AMD in lower-income groups rather than a true increase in disease prevalence. Conversely, socioeconomic determinants such as diet, smoking, and comorbid vascular disease may genuinely elevate AMD risk in these populations. Future population-based studies are warranted to disentangle the relative contributions of these factors.

## Strengths and limitations

This study possesses several important strengths. It is among the few investigations to assess referable AMD prevalence within a racially and socioeconomically diverse urban primary care cohort, thereby providing valuable real-world data on disease burden in an underserved population. The application of standardized Beckman Initiative grading criteria ensures comparability with major epidemiologic studies, while the large sample size and high image capture rate enhance the precision and reliability of prevalence estimates. Furthermore, linkage of clinical findings to area-level socioeconomic data enabled the quantification of significant income-related disparities in AMD risk. Some limitations of this study include the following: The single-center, urban safety-net clinic setting may limit the generalizability of findings to other populations, particularly those in rural or higher-income areas. SES was approximated using ZIP code–linked census data, which may not fully capture individual-level variability. The study cohort represents a convenience sample from a single hospital-based primary care clinic with a modest participation rate of 27.1%. Some patients declined participation due to time limitations or competing medical priorities. While such factors may introduce selection bias related to healthcare access or engagement, participants and non-participants did not differ significantly in age, sex, or race/ethnicity, suggesting reasonable representativeness of the clinic population. Nevertheless, because the study was based on a convenience sample from a single clinic rather than population-based sampling, the results should be interpreted as reflecting sample prevalence rather than population prevalence. The use of a convenience sampling approach limits generalizability to broader populations and may introduce selection bias related to healthcare-seeking behavior or comorbidities among participants. Additionally, our analysis did not include several known individual-level risk factors for AMD, such as smoking status, hypertension, diabetes, and body mass index, as these data were not consistently available in the EHR for all participants. The absence of these variables may have introduced residual confounding, as these conditions are associated both with AMD risk and with SES. Future studies incorporating detailed clinical and lifestyle risk factors will be important to more fully elucidate the independent contribution of social determinants to AMD prevalence.

## Electronic Supplementary Material

Below is the link to the electronic supplementary material.

Supplementary Material 1.

## Data Availability

The datasets generated and/or analyzed during the current study are not publicly available due to institutional policies protecting patient privacy and confidentiality. Researchers may contact the corresponding author for further information.
