# Supplementary material for: Elevated prevalence of age-related macular degeneration in a low-income urban primary care setting
Source: Discov Public Health. 2026 Mar 14;23(1):321. doi: 10.1186/s12982-026-01672-0 (PMC12989009; doi:10.1186/s12982-026-01672-0)

**Supplementary Tables**

**Table S1:** Univariate Logistic Regression Analyses for AMD by Demographic and Socioeconomic Predictors


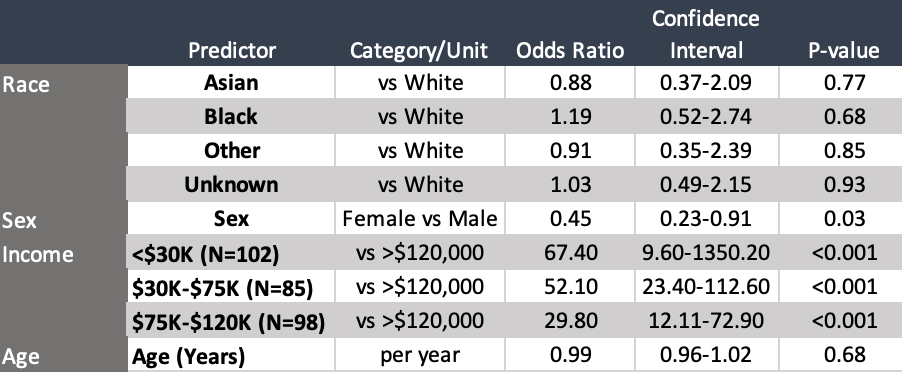


**Table S2:** Collinearity Diagnostics and Variable Contributions for Predictors of AMD


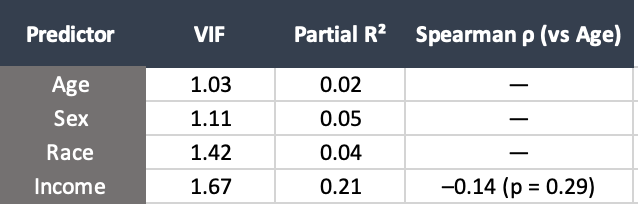

Supplement: Supplementary file 1 — Supplementary Material 1. [file 12982_2026_1672_MOESM1_ESM.docx]
